# Predictors of Chronic Ankle Instability Among Soccer Players

**DOI:** 10.3390/medicina61040555

**Published:** 2025-03-21

**Authors:** Ahmad Alanazi

**Affiliations:** Department of Physical Therapy and Health Rehabilitation, College of Applied Medical Sciences, Majmaah University, Al Majmaah 11952, Saudi Arabia; aalanazi@mu.edu.sa

**Keywords:** chronic ankle instability, soccer players, training hours, Ar-CAIT, sports rehabilitation

## Abstract

*Background and Objectives*: Chronic ankle instability (CAI) is prevalent among soccer players, often resulting from recurrent ankle injuries (RAIs). Despite its impact on performance and long-term joint health, the associated risk factors remain insufficiently explored. This study aimed to identify the key risk factors for CAI among soccer players. *Materials and Methods*: A cross-sectional study was conducted among 310 soccer players from different professional sports clubs. The Arabic version of the Cumberland Ankle Instability Tool (Ar-CAIT) was used to assess ankle instability. Spearman’s rho correlation and multiple linear regression were used to identify significant predictors of CAI. Additionally, structural equation modeling (SEM) was used to conduct mediation analysis and evaluate potential indirect effects. *Results*: Spearman’s correlation analysis revealed significant negative associations between Ar-CAIT scores and both BMI (*r* = −0.158, *p* < 0.05) and RAI (*r* = −0.273, *p* < 0.01), while training hours were positively correlated with Ar-CAIT scores (*r* = 0.169, *p* < 0.05). Multiple regression analysis confirmed that higher BMI (β = −0.133, *p* = 0.017) and a greater number of ankle injuries (β = −0.285, *p* < 0.001) were associated with lower Ar-CAIT scores, whereas increased training hours (β = 0.140, *p* = 0.010) were predictive of better ankle stability. Mediation analysis revealed that BMI and training hours partially mediate the relationship between RAI and Ar-CAIT scores. *Conclusions*: RAI, elevated BMI, and reduced training hours were significant predictors of CAI in soccer players. These findings emphasize the importance of implementing targeted injury prevention and rehabilitation strategies, particularly focusing on weight management and structured training programs to reduce CAI risk. Future longitudinal studies are required to explore the underlying mechanisms contributing to CAI development.

## 1. Introduction

Ankle injuries are prevalent among athletes, accounting for up to 30% of all sports-related injuries [1]. Repeated ankle injuries can lead to the development of chronic ankle instability (CAI), which is characterized by repeated episodes of the ankle “giving way”, diminished proprioception, and persistent pain or discomfort [2,3]. Sports such as soccer, basketball, and volleyball require athletes to perform different movements (e.g., changing direction, cutting, and running) at higher speeds, which may place them at higher risk of recurrent ankle injuries and consequently lead to CAI [4]. In a previous study, researchers identified impaired balance control and diminished ankle proprioception in athletes with CAI [4]. CAI significantly affects athletic performance and increases the risk of future injuries [5]. Additionally, athletes with CAI may experience longer recovery periods, reduced performance, and, consequently, shorter careers [6]. In professional athletes, ankle instability affects performance on the field, as pain, swelling, or fear of reinjury may hinder their ability to compete effectively [7]. Additionally, the psychological impact of ankle instability cannot be ignored [2]. Over time, this instability can lead to more serious consequences, such as osteoarthritis, muscle weakness, and long-term disability [8]. In addition to recurrent ankle injuries, other lower limb issues such as flexibility deficits and strength imbalances may contribute significantly to the risk of developing chronic ankle instability (CAI). For instance, flexibility limitations, particularly hamstring tightness, have been observed among younger soccer players and could potentially influence lower extremity mechanics and injury risk [9]. Furthermore, restricted range of motion in various lower limb joints has also been linked to increased injury occurrence in elite soccer academies, highlighting that flexibility and strength issues extend beyond the hamstring muscles alone [10].

The ankle joint is essential for stability, support, and adapting to rapid changes in direction and speed [11]. Mechanical and functional instability of the ankle often develops following an acute ankle sprain due to damage to the ligaments, muscles, and tendons surrounding the joint [12]. This damage can impair the ability of the ankle joint to stabilize during movement, increasing the risk recurrent injuries [13]. Recent research in the field of sports medicine has increasingly focused on the multifaceted nature of chronic ankle instability (CAI), particularly among soccer players [14,15]. It is essential to address a critical gap in the literature by identifying the interplay between various factors that are essential for the management of CAI. Numerous factors have been identified as predictors of chronic ankle instability, including the severity of the initial injury [16], the rehabilitation protocol [17], and the individual’s physical characteristics, such as body mass index (BMI), height, and age [18]. A higher BMI places increased stress on the ankle joint, causing greater instability [19]. Age can affect proprioception and balance, with older athletes potentially experiencing reduced ankle stability [20]. Additionally, training intensity and duration impact ankle function [21], and inadequate rehabilitation or overtraining can increase instability risk [22]. Various tools assess ankle-injury-related impairment, including the Foot and Ankle Ability Measure [23] and Tampa Scale for Kinesiophobia [24], which consider functional and psychological aspects. However, tools such as the Cumberland Ankle Instability Tool (CAIT) specifically assess the mechanical and functional instability of the ankle [25].

### Bottom of Form

The CAIT is a targeted and reliable self-reported measure for evaluating CAI [26] and has demonstrated high reliability and validity among elite athletes [27]. Athletes frequently experience ankle injuries, and the CAIT provides a quick, reliable, and validated way to assess the functional limitations caused by instability. Since the CAIT is self-reported, it captures an athlete’s own perception of ankle stability during high-performance activities [28]. Additionally, the CAIT is ideal for regular assessments in professional sports settings [29].

This study aimed to investigate key determinants of CAI in soccer players, focusing on factors such as history of injury, BMI, training hours, and physical characteristics. It was hypothesized that players with a history of repeated ankle injuries will exhibit significantly lower ankle stability, whereas a higher BMI will be associated with reduced stability. Conversely, increased training hours were expected to positively influence ankle stability.

## 2. Materials and Methods

### 2.1. Study Design and Setting

This cross-sectional study utilized a questionnaire survey to assess CAI. The Arabic version of the Cumberland Ankle Instability Tool (Ar-CAIT) was distributed once among the soccer players who meet the inclusion criteria. This study was approved by the Majmaah University Research Ethics Committee (MUREC-Oct 25/COM-2023/31-3). The study was carried out across different professional soccer clubs in the Riyadh province of Saudi Arabia. Soccer clubs were contacted, and a detailed explanation of the study protocol was provided to facilitate data collection.

### 2.2. Participants and Sampling Strategy

Participants were professional male soccer players aged 14 years or older, registered with their respective clubs for at least one year. A two-stage sampling approach was employed: initially, all 17 professional soccer clubs within Riyadh were contacted, and subsequently, 11 clubs responded positively. A total of 312 players were randomly selected from these 11 clubs through two-stage cluster sampling, stratified to ensure adequate representation across age groups and competitive levels. Players who had undergone ankle surgery, had significant lower limb injuries other than ankle sprains, or were diagnosed with medical conditions affecting joint stability were excluded. Two players withdrew due to health concerns, resulting in a final sample of 310 participants.

### 2.3. Inclusion and Exclusion Criteria

Participants were professional male soccer players, aged 14 years or older and with or without CAI, who were registered with a professional soccer club in Riyadh province. Players no longer actively engaged in soccer or who did not meet the practice requirements were excluded. Individuals with a history of surgical intervention for ankle instability were not included. Additionally, players who had sustained a significant lower limb injury or had known medical conditions affecting ankle stability were excluded. Furthermore, players who were unable or unwilling to provide informed consent or complete the questionnaire were not included in the study.

### 2.4. Sample Size

The required sample size was calculated based on 600 professional players from 17 soccer clubs, using a margin of error of 4%, a 95% confidence level, and a response distribution of 50%. According to the Raosoft sample size calculator and the specified parameters, the recommended minimum sample size was 301 players. This ensured statistically reliable results with the desired margin of error and confidence level while representing players across different age groups in the clubs.

### 2.5. Outcome Variable

The Ar-CAIT is a validated and user-friendly tool for assessing chronic ankle instability that is capable of differentiating between stable and unstable ankles. The original CAIT, developed in English by Hiller et al. (2006) [30], has demonstrated high content validity and test–retest reliability. The CAIT has been translated into several languages, including Brazilian Portuguese [31], Persian [32], Arabic [33], Korean [34], Japanese [35], Dutch [36], Greek [37], Chinese [38], Spanish [39], and French [40]. Different versions of the CAIT have been tested for internal consistency, test–retest reliability, ceiling and floor effects, and responsiveness. Korakakis et al. (2019) [33] demonstrated that the Ar-CAIT had robust psychometric properties, with a Cronbach’s alpha of 0.89, indicating high internal consistency, and an intraclass correlation coefficient (ICC) of 0.95, confirming excellent test–retest reliability. The questionnaire is widely understood throughout the Arabic-speaking world and can be utilized in research and clinical practice to evaluate patients with chronic ankle instability, where 0 point indicates extreme CAI and 30 points indicate a stable ankle [33]. The original study by Hiller (2006) [30] used a cutoff score of <27, while Wright et al. (2014) [41] suggested that a cutoff score of ≤25 enhances the tool’s accuracy in differentiating those with and without CAI.

### 2.6. Data Collection and Procedure

**Figure 1:**
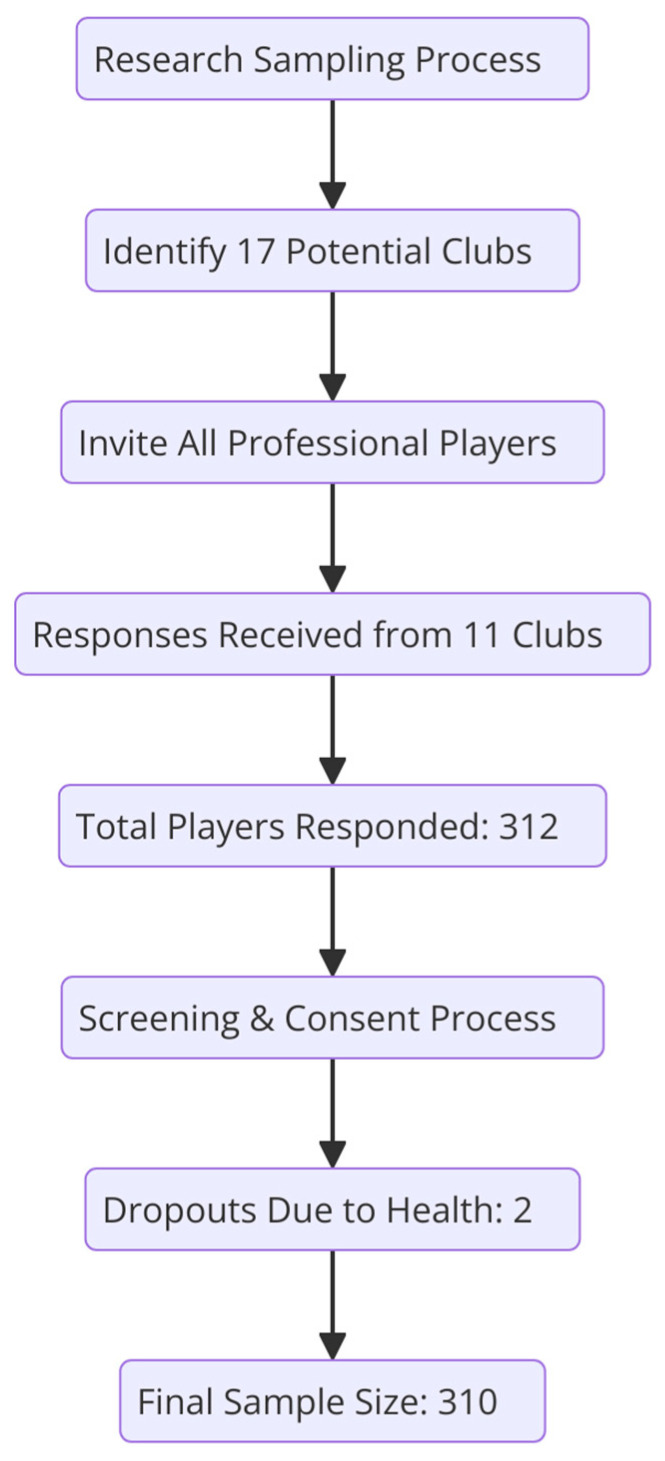
Flowchart showing sampling, recruitment, and response rates.

The procedures employed in this study were meticulously structured and adhered to academic conventions. The Saudi Arabian Soccer Federation was consulted to determine the appropriate sample size, resulting in a selection of 310 professional soccer players aged 14 years and older. All participants were professional male soccer players who met the inclusion and exclusion criteria. The study was conducted in different soccer clubs in Riyadh, utilizing a two-stage cluster sampling technique where players from all the clubs were clustered initially as a first stage and then 312 players were randomly selected and asked to fill the questionnaire. Written informed consent was obtained from all participants, emphasizing voluntary participation, confidentiality, and data privacy. All demographic risk factors, including players’ age, height, weight, BMI, dominant limb, play-related training hours, training years, history of initial injury, and recurrent ankle injuries were obtained using a structured form in Arabic. Anthropometric data including height, weight, and additional parameters were collected using the Seca 213 stadiometer and Seca 813 digital scale and all measurements were taken in the morning before starting training sessions with players wearing shorts and a T-shirt and without shoes. After obtaining the initial data, the Ar-CAIT was distributed among the 312 players for separate assessment of both the left and right ankles. Participants were given the choice to complete the questionnaire either online or onsite using a pen-and-paper version, according to their convenience. Regardless of their preferred method, each player received a detailed, face-to-face explanation of the questionnaire sections, including the rationale behind each part, provided by trained research assistants. To ensure the accuracy and authenticity of responses, online submissions were monitored closely by providing unique participant identifiers and preventing multiple entries from the same respondent. Completed questionnaires were handled confidentially, and data privacy was strictly maintained throughout the process. Among the 312 players, a 99% response rate (n = 310) was achieved, with two players withdrawing due to health issues (Figure 1).

### 2.7. Statistical Section

Statistical analysis was conducted using JASP 0.18.1. Due to non-normality among variables, Spearman’s rho correlation was employed to examine the relationships between AR-CAIT, age, height, weight, BMI, training duration, and recurrent ankle injuries (RAIs). Prior to regression analysis, assumptions of linearity and homoscedasticity were checked via scatterplots of standardized residuals against predicted values, which confirmed adequate model assumptions. Collinearity diagnostics indicated acceptable Variance Inflation Factors (VIFs) for all predictors, with the highest VIF observed for BMI (1.079); height and weight were excluded from further analysis due to multicollinearity with BMI. Mediation analysis was performed using structural equation modeling (SEM) in JASP, with indirect effects estimated via a bootstrapping procedure of 5000 resamples and bias-corrected confidence intervals. All SEM assumptions—including normality, linearity, absence of multicollinearity, and sufficient sample size—were met, and model fit was evaluated using RMSEA (≤0.08), CFI (≥0.90), and TLI (≥0.90). Mediation was considered present when the confidence intervals for the indirect effects did not include zero. Three analyses were conducted to ensure comprehensive understanding: correlation analysis to assess basic relationships, regression analysis to quantify the independent contributions of predictors such as BMI, training hours, and number of RAIs, and mediation analysis to explore the indirect pathways influencing AR-CAIT scores. The level of significance was set at *p* < 0.05.

## 3. Results

Table 1 presents the descriptive statistics for the Ar-CAIT scores, age, height, weight, BMI, training years, training hours, and RAI. The mean Ar-CAIT score was M = 18.6 (SD = 5.6), with an average participant age of M = 17.4 years (SD = 3.8). The mean height and weight were M = 169.1 cm (SD = 8.1) and M = 65.8 kg (SD = 10.8), respectively, while the mean BMI was M = 22.9 (SD = 3.2). Participants had been training for an average of M = 5.3 years (SD = 3.01), with weekly training hours averaging M = 2.1 h (SD = 0.95).

The Spearman correlation table reveals significant negative associations between Ar-CAIT and both BMI (−0.158 *) and RAI (−0.273 **). The number of training hours shows a positive correlation with the Ar-CAIT score (0.169 *). BMI shows a strong positive correlation with weight (0.810 *), while it is negatively correlated with training hours (−0.211 *). Moreover, RAI is negatively correlated with training hours (−0.118 *) (Table 2).

Multiple linear regression analysis was performed to determine the predictors of Ar-CAIT scores. The model accounted for 13% of the variance in the Ar-CAIT scores (R^2^ = 0.13, Adjusted R^2^ = 0.119, *p* < 0.001). The regression model was statistically significant (F [4, 305] = 11.431, *p* < 0.001), indicating that the predictors collectively contributed to explaining the variance in Ar-CAIT scores. Significant predictors included: BMI (β = −0.133, *p* = 0.017), training hours per week (β = 0.14, *p* = 0.01), and RAI (β = −0.285, *p* < 0.001). The standardized coefficients (β) indicate that the RAI variable had a moderate-to-large negative impact on ankle stability, whereas BMI and training hours showed small-to-moderate influences. Higher BMI and more frequent ankle injuries were associated with lower AR-CAIT scores, whereas increased training hours were associated with higher Ar-CAIT scores (Table 3).

### Mediation Analysis

**Figure 2:**
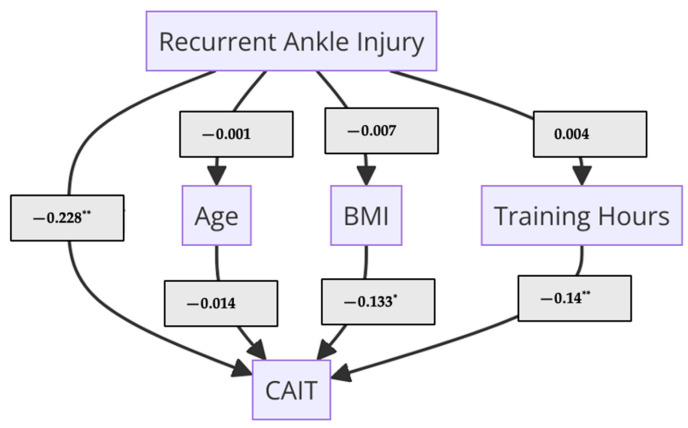
Mediation flow diagram. * *p* < 0.05 and ** *p* < 0.01 are statistically significant; CAIT, Cumberland Ankle Instability Tool; BMI, body mass index.

Mediation analysis was conducted to assess the impact of the RAI on Ar-CAIT scores, considering potential mediators such as age, BMI, and training hours. The results revealed that RAI had a significant direct negative effect on Ar-CAIT scores (Estimate = −0.228, 95% CI [−0.323, −0.137], *p* < 0.001), indicating that a higher frequency of ankle injuries substantially reduced Ar-CAIT scores. However, none of the indirect effects through age, BMI, or training hours were statistically significant (e.g., RAI → BMI → Ar-CAIT: Estimate = −0.007, *p* = 0.272, 95% CI [−0.029, 0.002]), suggesting that these mediators did not substantially mediate the relationship between RAI and Ar-CAIT scores. Significant path coefficients indicated that a higher BMI negatively affected the Ar-CAIT score (Estimate = −0.133, *p* = 0.016, 95% CI [−0.244, −0.022]), whereas increased training hours positively influenced Ar-CAIT scores (Estimate = 0.14, *p* = 0.009, 95% CI [0.028, 0.253]). Additionally, residual covariances indicated significant associations between BMI and training hours (Estimate = −0.183, *p* = 0.001, 95% CI [−0.31, −0.063]). The model explained 13% of the variance in Ar-CAIT scores (R^2^ = 0.13), highlighting the direct role of injuries and the independent contributions of BMI and training hours to Ar-CAIT score (Figure 2).

## 4. Discussion

The aim of this study was to investigate the influence of the number of recurrent injuries, BMI, training hours, and age on CAI among elite athletes. The primary findings indicated that RAI, BMI, and number of training hours significantly impacted ankle instability.

The results of the study indicate that recurrent ankle injuries are a significant predictor of ankle instability in professional soccer players. These findings align with previous research showing that repeated ankle sprains compromise joint stability and proprioception, contributing to ankle instability [42,43]. This reinforces the understanding that ligament damage from repeated injuries reduces the mechanical and functional stability of the ankle (altered muscle activation) [44], creating a vicious cycle of increased instability and, consequently, a higher risk of ankle injury [45]. Similar findings have been reported in studies examining recurrent ankle sprains and chronic instability in both general and athletic populations [16,46,47,48].

In this study, a higher BMI was found to be associated with higher ankle instability. However, the positive effect of training hours on Ar-CAIT scores was partially mediated by BMI, implying that a better body composition may enhance the benefits of training. This finding is supported by studies that highlight the detrimental effects of excess body weight on joint health [49], particularly in weight-bearing joints such as the ankle. Increased mechanical load due to a higher BMI places greater strain on the musculoskeletal system, contributing to injury and instability. These results are consistent with previous research linking a higher BMI to an increased risk of musculoskeletal injuries [50] and sensorimotor dysfunction [51].

Furthermore, the findings suggest that longer training hours improve ankle stability, potentially due to the positive effects of exercises on ankle proprioception, muscle strength, and muscle activation [52,53]. Regular and structured training likely enhances neuromuscular control, proprioception, and muscle strength, thereby reducing the risk of recurrent injuries. In this study, while an association between training hours and injury risk/recovery outcomes was found, the reliance on self-reported data may have introduced biases such as recall error and the underreporting of injury histories. Future research should incorporate objective measures, such as wearable technology or verified training logs in an effort to provide a more accurate assessment of these relationships. This is consistent with studies emphasizing the role of targeted proprioceptive and balance training in improving joint stability and reducing RAI risk [54]. Conditioning programs targeting the ankle after injury are designed to mitigate the effects of prior injuries, thereby improving stability. However, some studies reported contradictory findings, suggesting that excessive training without sufficient recovery may elevate the risk of overuse injuries [55,56,57]. These inconsistencies could be attributed to differences in training quality and the extent to which preventive exercises are incorporated into rehabilitation protocols, emphasizing the importance of a well-balanced training regimen [58,59].

Age did not significantly impact Ar-CAIT scores, which was somewhat unexpected. Older athletes and those with more years of experience may be more prone to signs of wear and tear or an age-related decline in proprioception. However, this lack of significance could be explained by the relatively young mean age of the participants (17.5 years). Moreover, effective injury management and conditioning programs can help maintain joint stability despite aging. Similarly, other studies have found that while older athletes may have higher injury risks, proper rehabilitation and maintenance programs can mitigate the effects of aging on joint function [60,61].

### Limitations

This study has several limitations. First, it relied on self-reported measures of ankle instability using the Ar-CAIT, which could introduce recall bias and subjective interpretation by participants. Athletes might have overestimated or underestimated their symptoms based on personal perceptions or recent experiences, potentially affecting the accuracy of the results. Such inaccuracies could affect the validity and precision of the observed associations. Therefore, future studies should complement subjective assessments with objective measures, such as gait analyses, electromyography, force platforms, or biomechanical assessments to provide a more comprehensive and objective evaluation of chronic ankle instability. Also, future studies are recommended to combine self-reported measures with objective clinical or biomechanical assessments (e.g., functional testing, strength testing, or sensor-based measures) to enhance data validity and reduce potential reporting biases. Second, the design of the study limited the ability to establish causal relationships between the analyzed variables and chronic ankle instability. Although the regression analysis identified significant associations among recurrent injury, BMI, training hours, and ankle stability, it could not determine the direct cause-and-effect relationship. Longitudinal studies are necessary to track changes in ankle stability over time and to understand how factors such as training interventions or injury prevention strategies influence long-term outcomes. Finally, the study sample was limited to soccer players, which may restrict the generalizability of the findings to other populations. These results may not apply to recreational athletes or the general population, who may experience different injury patterns, rehabilitation, and recovery. Additionally, this study did not include female athletes and did not account for potential influences of different playing surfaces, players undergoing strength training, playing position, footwear and protective equipment used, or previous treatments and rehabilitation protocols received, which might influence chronic ankle instability outcomes.

## 5. Conclusions

This study demonstrated that repeated injuries, BMI, and training hours are significant factors influencing chronic ankle instability among professional soccer players. These findings are consistent with the existing literature on the detrimental effects of recurrent ankle sprains and excess body weight on joint stability, while also emphasizing the protective role of regular structured training. Understanding these factors is essential for developing personalized rehabilitation programs aimed at further reducing injury risk and enhancing ankle stability in professional athletes. Future research should employ longitudinal study designs to clarify causality, monitor ankle instability progression, and evaluate the long-term outcomes of interventions. Additionally, future research should include biomechanical assessments and objective functional tests and explore other potential contributing factors, such as playing position and environmental influences, to develop comprehensive and tailored prevention and rehabilitation strategies.

## Figures and Tables

**Table 1:** Demographic data.

Variable	M	SE	95% CI [Lower, Upper]	SD
Ar-CAIT	18.6	0.31	[18.0, 19.2]	5.6
Age	17.4	0.21	[17.0, 17.9]	3.8
Height (cm)	169.1	0.46	[168.2, 170.0]	8.1
Weight (kg)	65.8	0.61	[64.6, 67.0]	10.8
BMI	22.9	0.18	[22.6, 23.3]	3.2
Training Years	5.3	0.17	[5.0, 5.6]	3.01
Training Hours	2.1	0.05	[2.0, 2.2]	0.95
RAIs	1.5	0.07	[0.07, 1.7]	1.2

Note: M, mean; SD, standard deviation; SE, standard error; CI, confidence interval; Ar-CAIT, Arabic version of Cumberland Ankle Instability Tool; BMI = body mass index; RAIs = recurrent ankle injuries.

**Table 2:** Spearman’s rho correlation between variables.

Variable	Ar-CAIT	Age	Height	Weight	BMI	Training Years	Training Hours
Age	−0.030						
Height	0.089	0.002					
Weight	−0.072	0.145	0.506 *				
BMI	−0.158 *	0.119	−0.034	0.810 *			
Training Years	0.087	0.388	0.001	0.077	0.049		
Training Hours	0.169 *	−0.032	0.096	−0.118 *	−0.211 *	−0.035	
RAIs	−0.273 **	0.079	0.044	0.122	0.107	0.049	0.009

Correlations marked with * *p* < 0.05 and ** *p* < 0.01 are statistically significant; Ar-CAIT, Arabic Version of Cumberland Ankle Instability Tool; BMI, body mass index; RAIs, recurrent ankle injuries.

**Table 3:** Regression coefficient with CAIT scores as the dependent variable.

					95% CI		Assumption Testing (<10)
Predictors	Unstandardized	Standardized	t	*p*	Lower	Upper	VIF
(Intercept)	18.652		59.455	<0.001	18.024	19.279	
(Intercept)	24.562		9.406	<0.001	19.423	29.700	
Age	−0.020	−0.014	−0.252	0.802	−0.178	0.137	1.054
BMI	−0.232	−0.133	−2.400	0.017	−0.423	−0.042	1.079
Training Hours	0.828	0.140	2.578	0.010	0.196	1.460	1.038
RAIs	−1.283	−0.285	−5.302	<0.001	−1.759	−0.807	1.016

CAIT, Cumberland Ankle Instability Tool; BMI, body mass index; RAIs, recurrent ankle injuries.

## Data Availability

Data used in this study are available from the corresponding author upon reasonable request.
